# Molecular Evolution of *PTEN* Pseudogenes in Mammals

**DOI:** 10.1371/journal.pone.0167851

**Published:** 2016-12-09

**Authors:** Jingsi Tang, Ruihong Ning, Bo Zeng, Ying Li

**Affiliations:** Farm Animal Genetic Resources Exploration and Innovation Key Laboratory of Sichuan Province, Sichuan Agricultural University, Chengdu, China; University of Florida, UNITED STATES

## Abstract

Phosphatase and tensin homolog (*PTEN*) is a tumor-suppressor gene. *PTEN* pseudogene (*PTENp*) acts as an endogenous RNA, which regulates its parental gene by competitively binding to the 3’ UTR of *PTEN* gene in the human. Despite the importance of this pseudogene, little is known about the molecular evolution of *PTENp* in mammals. In this study, we identified 37 pseudogenes from 65 mammalian genomes. Among them, 32 were from rodents or primates. Phylogenetic analyse showed a complex evolutionary history of this gene family. Some *PTENps* were shared both in primates and rodents. However, some *PTENps* were shown to be species-specific, such as the tasmanian devil *PTENp*1, nine banded armadillo *PTENp*1 and gibbon *PTENp*1. Most interestingly, the naked mole rat (NMR), an anticancer model organism, possessed 17 copies of *PTENps*, which were classified into four clades based on the phylogenetic analyses. Furthermore, we found that all the 3’UTR of *PTEN* and *PTENps* shared common microRNA (MicroRNA) binding sites in NMR, based on our prediction of specific MicroRNA binding sites. Our findings suggested that multiple gene duplications have occurred in the formation of PTEN/PTENp gene family during the evolution of mammals. Some *PTENps* were relatively ancient and were shared by primates and rodents; others were newly originated through species- specific gene duplications. *PTENps* in NMR may function as competitive endogenous RNAs (ceRNAs) to regulate their counterpart genes by competing for common MicroRNAs, which may be one of the interpretations for the cancer resistance in NMR.

## Introduction

In 1977, Jacq et al found a truncated version of the 5S ribosome DNA in *Xenopus laevis*, which is homologous to the native gene, and this fragment of genomic sequences was first named Pseudogene[1]. Traditionally, pseudogenes were defined as the functionless relatives of protein-coding genes, mainly due to the presence of premature stop-codons or frame shifts, and have long been viewed as the non-functional genomic remnants during evolution[2]. Based on their formation mechanisms, pseudogenes can be classified into three categories, which are unitary pseudogenes, unprocessed pseudogenes, and processed pseudogenes. Unitary pseudogenes, previously referred to those functionless genes, originated from functional genes by various mutations. Unprocessed pseudogenes are derived directly from duplications of DNA sequences, with their original intron-exon structures and promoter elements having been maintained. Processed pseudogenes are formed by retrotransposition of mRNA transcripts. Introns and other regulatory elements such as enhancers and promoter elements have been lost during the process of pseudogenization.

It is proposed that messenger RNA, transcribed pseudogenes, and long non-coding RNAs can crosstalk by competing for common MicroRNAs[3,4]. These RNA transcripts were termed as competitive endogenous RNAs (ceRNAs).The activity of ceRNAs forms a large-scale regulatory network across the transcriptome. More and more experimental evidences, such as *PTEN-PTENP1*[5], *TUSC2-TUSC2P*[6], *HMGA1-HMGA1Ps*[7], *CYP4Z1-CYP4Z2P*[8] and *BRAF-BRAFP1*[9], support the ceRNA regulation hypothesis. For example, PTEN negatively regulates intracellular levels of phosphatidylinositol-3,4,5-trisphosphate in cells and acts as a tumor suppressor by negatively regulating Akt/PKB signalling pathway[10].The PTEN pseudogene (PTENp1) is a processed pseudogene, which shows high sequence similarity with *PTEN* in human. The binding sites of the MicroRNAs, including miR-20a, miR-19b, miR-21, miR-26a and miR-214, are highly homologous in the 3’UTR of *PTEN* and *PTENp1*, and those MicroRNAs are able to regulate the translation of *PTEN* in humans[5]. *PTENp1* can thus regulate *PTEN* by competitively binding to these MicroRNAs, and serving as decoy for PTEN-related MicroRNAs. Furthermore, decreasing of the copy number of *PTENp1* was observed insporadic colon cancer, which was correlated with a decrease of *PTEN*, thus leading to the proposal that *PTENp1* is a bona fide tumour suppressor gene[5]. In addition, Johnsson et al. reported that PTENp1-expressed transcripts can also actasantisense RNAs (asRNAs) to regulate *PTEN* expression at both transcriptional and post-transcriptional levels[11]. *PTENp1* encoded two asRNA isoforms: *PTENp1* asRNA alpha and beta. The alpha isoform acts as a negative regulator for transcription of *PTEN*. Because of the sequence homology, *PTENp1* asRNA alpha recruits the DNA methyltransferase 3a (DNMT3a) and Enhancer of Zeste Homolog 2 (EZH2) to the *PTEN* promoter, resulting in *PTEN* transcription suppressed by the formation of H3K27me3. In contrast, the beta isoform forms RNA-RNA interactions with *PTENp1* sense transcript. This RNA-RNA interaction stabilizes *PTENp1* sense, consequently affecting MicroRNA sequestration and ultimately *PTEN* protein level.

Except for *PTENp1*, some other pseudogenes were reported to perform as ceRNAs. The tumour suppressor candidate-2 gene pseudogene (TUSC2P) can talk with the TUSC2 gene through MicroRNA response elements (MREs), as well as PTEN-PTENP1. The3’UTR of *TUSC2P* captures these TUSC2-targeting MicroRNAs, which increases the translation of *TUSC2* and then inhibits cell proliferation[6]. In addition, Esposito and co-workers found seven pseudogenes homologous to the high mobility group AT-hook 1 (HMGA1) gene, which is associated with insulin resistance and carcinogenesis[7]. Two of them, the *HMGA1P6* and *HMGA1P7*, showed high sequence similarity with each other and conserved MRE with the parental gene. *HMGA1P6* and *HMGA1P7* also act as ceRNAs by competitively binding to MicroRNAs with the *HMGA1*, regulating the expression of *HMGA1* and accordingly increasing proliferation and cell migration[7]. Florian et al. discovered that *BRAFP1* functions as a ceRNA of *BRAF* in humans and mice, competing for miR-134, miR-543, miR-653, miR-30a, miR-182 and miR-876[9]. Most interestingly, the effect of over-expression of the 3’UTR of *BRAFP1*was more significant than over-expression of its CDS on the parental gene expression and proliferation[9]. Overall, these findings suggest that 3’UTRs from both pseudogenes and coding genes may possess powerful biological activity through their ability to act as endogenous decoys for MicroRNAs.

Despite the importance of those functional pseudogenes, their evolutionary histories were largely unknown. In this study, we investigated the molecular evolution of PTEN/PTENp gene family in mammals. By searching the available mammalian genome sequences, we found 37 pseudogenes from 65 mammalian genomes. Most intriguingly, we identified 17 copies of *PTENps* from naked mole rat (NMR), an anticancer model organism, and found that all of these genes shared common MicroRNA binding sites with their PTEN gene, suggesting that the *PTENps* in NMR may be functional in regulating their cognate genes by competing for MicroRNA binding sites, just as that found in the humans.

## Materials and Methods

Our animal experiment was approved by the Institutional Animal Care and Use Committee of the Sichuan Agricultural University under permit number DKY- B20150301

### Sequences obtain

The *PTEN* mRNA sequences from 65 mammals were downloaded from National Centre for Biotechnology Information (NCBI) GenBank, and their *PTENps* were identified by BLAST, the reference genomic sequences database, using *PTEN* mRNA as query. All potential pseudogenes meet at least one of the following three criterions:1. incomplete open reading frame (ORF), 2. frame-shifts and 3. premature stop codons. All were labelled as pseudogenes in GenBank.

### Phylogenetic analyses

As different regions of a gene play different roles and are, apparently, subjected to different stringencies of functional constraints, it has been customary to treat different regions separately. In contrast to the coding regions of genes, the rates in non-coding regions are usually higher. Furthermore, most of them vary greatly in the length of these noncoding regions. For example, the length of 3’UTR of *PTEN*/*PTENps* in primates are largerat1000bp, but in most of other species are less than 1000bp.This variation makes the phylogenetic analyses using noncoding regions very difficult.Therefore, in this study, we only compared the evolutionary rate of CDS of *PTEN*/*PTENps*. The CDS region of *PTEN* and *PTENp* sequences of mammals were aligned using ClustalW in BioEdit[12] followed by manual adjustments. Maximum Likelihood (ML), Maximum Parsimony (MP) and Neighbour Joining (NJ) phylogenetic trees were conducted by using MEGA6.0[13]. Fourteen sequences out of 102 identified *PTEN*s and *PTENps* were removed in the phylogeny analyses because of too many ambiguous bases, long gaps or the incompleteness of the sequences. The removed sequences were degu *PTENp1*, NMR*PTENp12*, NMR *PTENp15*, NMR *PTENp16*, horse *PTEN*,orangutan*PTENp1*and chimpanzee *PTENp1* (these sequences contained ambiguous bases); cattle *PTEN*, duckbill platypus *PTEN*, domestic water buffalo *PTEN*, southern American pika*PTEN* and orguinea pig *PTENp1* (these sequences showed big gaps); guinea pig *PTEN* and European domestic ferret *PTEN* (these sequences were incomplete). And then the coding regions of 88 sequences were used for phylogenetic tree construction. In addition, a dataset contains 67 sequences from Primate, Rodents, Even-toed ungulates, Carnivores, Cingulata and Dasyuromorphia, where both *PTEN* and *PTENps* were identified, was also used to construct phylogenetic trees. Kimura 2-parameter method[14] were used to infer NJ tree implemented in the program MEGA6.0. For ML tree, Tamura 3-parameter model with a discrete gamma distribution was used as suggested by MEGA6.0. Default settings in MEGA6.0 were used in reconstructing the MP tree. For ML, MP and NJ methods, 1000 bootstrap replications were conducted to evaluate the reliabilities of the reconstructed trees.

### MicroRNAs binding sites Prediction

MicroRNA binding sites of the 3’UTR of *PTENs* and *PTENps* were predicted by PITA algorithm[15]. Five MicroRNAs (mir-19b, mir-20a, mir-21, mir-26a, mir-214),which can competitively bind with *PTEN* and *PTENp*1 in human[5], were downloaded from miRBase database[16]. Then the specific MicroRNAs and 3’ UTR of *PTENs* or *PTENps* were uploaded to the Online microRNA prediction tool to predict MicroRNA binding sites[15]. Minimum seed size is set to 6 and other parameters were as default settings. ΔΔG is an energetic score, the lower (more negative) its value, the stronger the binding of the MicroRNA to the given site is expected. We first calculated the ΔΔG values for the known binding pairs of MicroRNA and the corresponding binding sites in the humans, and then conservatively set the lowest value-3.8 as our cut-off value in this study.

## Results

### Pseudogene Sequences

In total, we found 65 functional genes and 37 pseudogenes from 65 genomes of mammals by BLAST using *PTEN* mRNA as query (S1 and S2 Tables). We found that 17 out of 65 species possess one or more copies of *PTENps*. Among them, 32 out of 37 pseudogenes identified in this study were from primates and rodents. We identified 9 species each possessed one *PTENp* in primate. Interestingly, these *PTENps* only existed in old world monkeys and hominoids. Five species of rodents were found to possess *PTENps*. And most excitingly, 17 copies of *PTENps* in NMR were identified (Table 1). In addition, one copy of *PTENp* was found in the nine banded armadillo and Tasmanian devil. Three copies of *PTENps* were found in the pig.

**Table 1:** The number of *PTENs* and *PTENps* in mammals.

Order	Common name	Scientific name	No. of PTEN	No. of PTENp
Primates	Human	Homo sapiens	1	1
	Chimpanzee	Pan troglodytes	1	1
	Pygmy chimpanzee	Pan paniscus	1	1
	Orangutan	Pongo abelii	1	1
	Gorilla	Gorilla gorilla gorilla	1	1
	Crab-eating macaque	Macaca fascicularis	1	1
	Rhesus macaque	Macaca mulatta	1	1
	Baboon	Papio anubis	1	1
	Green monkey	Chlorocebus sabaeus	1	NO
	Marmoset	Callithrix jacchus	1	NO
	Squirrel monkey	Saimiri boliviensis boliviensis	1	NO
	Gibbon	Nomascus leucogenys	1	1
	Bush baby	Otolemur garnettii	1	NO
Rodents	Mouse	Mus musculus	1	NO
	Rat	Rattus norvegicus	1	NO
	Naked mole rat	Heterocephalus glaber	1	17
	Blind mole rat	Nannospalax galili	1	1
	Golden hamster	Mesocricetus auratus	1	NO
	Chinese hamster	Cricetulus griseus	1	NO
	Prairie vole	Microtus ochrogaster	1	NO
	Prairie deer mouse	Peromyscus maniculatus bairdii	1	NO
	Guinea pig	Cavia porcellus	1	1
	Lesser egyptian jerboa	Jaculus jaculus	1	NO
	Degu	Octodon degus	1	3
	Long-tailed chinchilla	Chinchilla lanigera	1	1
	Thirteen-lined ground squirrel	Spermophilus tridecemlineatus	1	NO
Even-toed ungulates	Bovine	Bos taurus	1	NO
	Wild yak	Bos mutus	1	NO
	Goat	Capra hircus	1	NO
	Sheep	Ovis aries	1	NO
	Chiru	Pantholops hodgsonii	1	NO
	Domestic water buffalo	Bubalus bubalis	1	NO
	Killer whale	Orcinus orca	1	NO
	Sperm whale	Physeter catodon	1	NO
	North Pacific minke whale	Balaenoptera acutorostrata scammoni	1	NO
	Pig	Sus scrofa	1	3
	Alpaca	Vicugna pacos	1	NO
	Wild bactrian camel	Camelus ferus	1	NO
Carnivores	European domestic ferret	Mustela putorius furo	1	NO
	Dog	Canis lupus familiaris	1	NO
	Cat	Felis catus	1	NO
	Siberian tiger	Panthera tigris altaica	1	NO
	Weddell seal	Leptonychotes weddellii	1	NO
	Pacific walrus	Odobenus rosmarus divergens	1	NO
	Giant panda	Ailuropoda melanoleuca	1	NO
Bats	Brandt's bat	Myotis brandtii	1	NO
	David's myotis	Myotis davidii	1	NO
	Little brown bat	Myotis lucifugus	1	NO
	Black flying fox	Pteropus alecto	1	NO
Odd-toed ungulates	Horse	Equus caballus	1	NO
	Southern white rhinoceros	Ceratotherium simum simum	1	NO
Insectivores	Eurasian common shrew	Sorex araneus	1	NO
	Star-nosed mole	Condylura cristata	1	NO
	Cape golden mole	Chrysochloris asiatica	1	NO
	Cape long-eared elephant shrew	Elephantulus edwardii	1	NO
	Lesser hedgehog tenrec	Echinops telfairi	1	NO
Rabbits & Hares	Southern American pika	Ochotona princeps	1	NO
	Rabbit	Oryctolagus cuniculus	1	NO
Tree shrew	Chinese tree shrew	Tupaia chinensis	1	NO
Sirenia	Florida manatee	Trichechus manatus latirostris	1	NO
Cingulata	Nine-banded armadillo	Dasypus novemcinctus	1	1
Proboscidea	African elephant	Loxodonta africana	1	NO
Dasyuromorphia	Tasmanian devil	Sarcophilus harrisii	1	1
Didelphimorphia	Gray short-tailed opossum	Monodelphis domestica	1	NO
Monotremes	Duckbill platypus	Ornithorhynchus anatinus	1	NO

### Phylogenetic analyses

**Fig 1:**
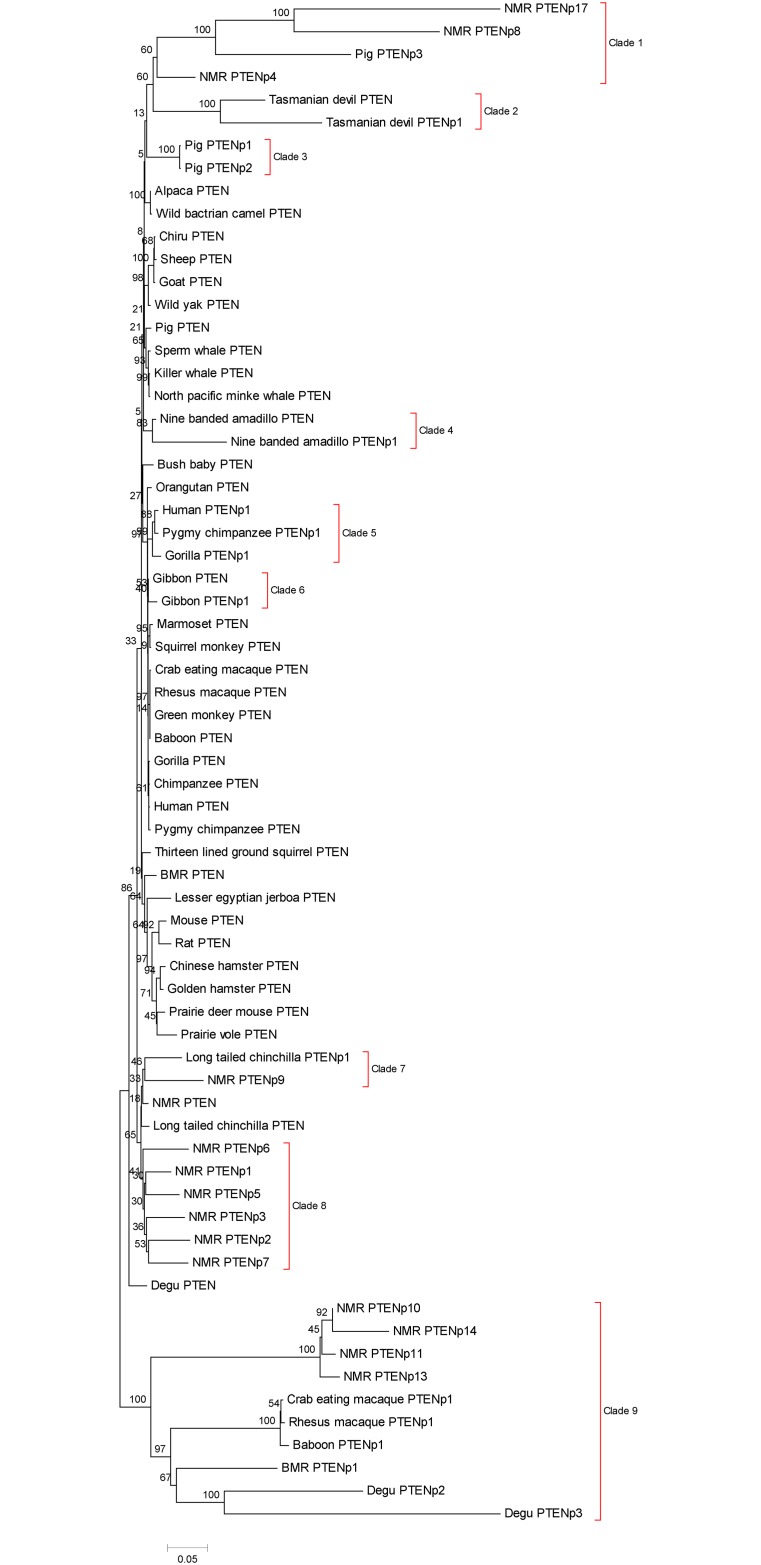
The Neighbour Joining tree of 67 *PTEN*s and *PTENps* in mammals. All bootstrap values were showed. The tree is drawn to scale, with branch lengths in the same units as those of the evolutionary distances.Clade 1 to clade 9 were tagged by red square.

To explore the evolutionary relationships of these PTENps and PTEN genes in mammals, we constructed the phylogenetic trees based on the coding region of 88 sequences using the Neighbour joining (NJ)[17](S1 Fig), Maximum parsimony (MP)[18](S2 Fig) and Maximum Likelihood (ML) methods (S3 Fig) separately. To make the result more clear, we removed sequences from those orders where no PTEN pseudogene was found. Based on the coding region of the remaining 67 sequences, we constructed the NJ (Fig 1), MP (S4 Fig) and ML tree (S5 Fig). All trees showed overall similar topology. In these trees, *PTENps* were dispersed into several clades rather than forming one clade, suggesting that multiple gene duplications have occurred during the evolution of PTEN/PTENp gene family (Fig 1 and S1–S5 Figs). As showed in Fig 1, some *PTENps* existed for a relatively long time such as clade 1, which was shared by the NMR and the pig; and clade 9,which was shared by species from primates and rodents. In addition, the two clades showed longer branch lengths compared to other clades of *PTENps*, which indicated that *PTENps* of the two clades were relatively old. However, some *PTENps* were relatively young. For example, clade 2, clade 4 and clade 6 displayed a species-specific evolutionary pattern, in which *PTENp* clustered with its cognate gene, suggesting these *PTENps* emerged after the divergences of these species from their sister groups. What’s more, we found that the branch lengths of *PTENps* were longer than that of the *PTEN*, suggesting faster evolutionary rate of CDS of *PTENps* than the *PTEN*s in mammals (Fig 1). *PTENps* in NMR were divided into four clades in the phylogenetic tree. Clade 1 includes *PTENp*17, *PTENp*8 and *PTENp*4. Clade 7 contains only PTENp9. Clade 8 includes *PTENp*1, *PTENp*2, *PTENp*3, *PTENp*5, *PTENp*6 and *PTENp*7. Clade 9 includes *PTENp*10, *PTENp*11, *PTENp*13 and *PTENp*14.*PTENps* in clade 8 were NMR specific with shorter branch lengths, suggesting that these genes appeared recently.

### MicroRNA binding sites prediction

**Fig 2:**
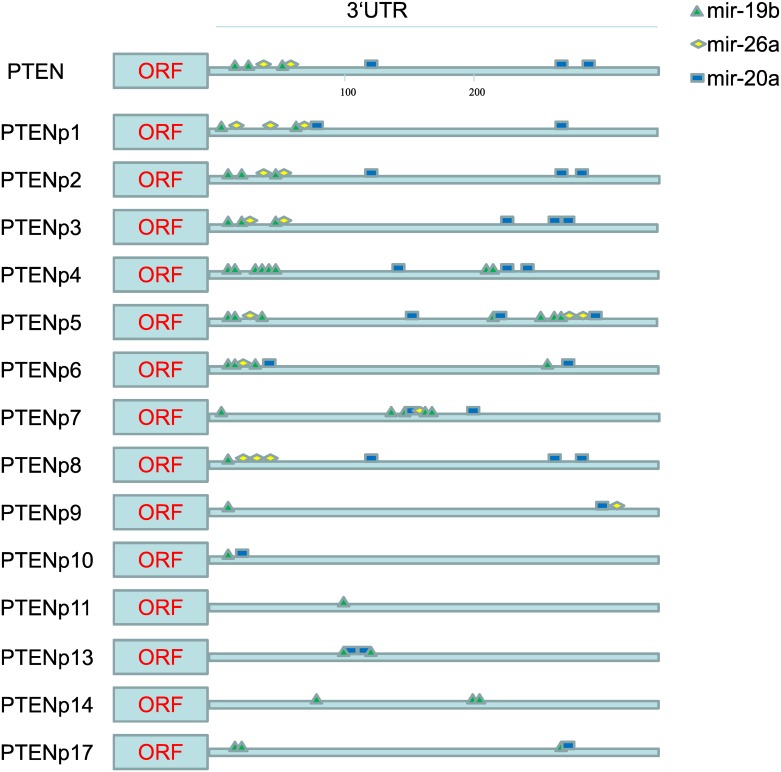
Shared MicroRNAs binding sites of the 3’UTR of *PTEN* and *PTENps* in NMR. The green triangle represents the mir-19b binding site, the yellow diamond represents the mir-26a binding site, and the blue rectangle represents the mir-20a binding site.

To investigate whether 3’UTR of these *PTENps* could potentially bind to specific MicroRNAs just like in the human, we used the PITA algorithm to predict the binding sties of specific MicroRNAs on 3'UTR of *PTENps*. We chose the PITA for MicroRNA target prediction because it has high prediction accuracy and low false positive rate, since it pays more attention to the accessibility but not the conservation of the target sequences[15,19]. It was evidenced that 5 MicroRNAs (mir-19b, mir-20a, mir-21, mir-26a, mir-214) could bind to the 3'UTR of *PTEN* and *PTENp*1, and thus act as MicroRNA sponges to protect their parent gene from MicroRNA disturbancein the human[5]. In this study, we predicted the binding sites of these five MicroRNAs in 3’UTR of *PTEN* and *PTENps* identified in this study (S3 and S4 Tables). Interestingly, we found that the 3’UTR of *PTENp* and *PTEN* shared MicroRNAs bind sites in most cases, except for the *PTENp*1 in the blind mole rat (BMR), in which no shared binding site was found. This result suggested that MicroRNAs were potentially able to bind to the 3' UTR of both *PTENps* and their cognate *PTEN*s. Most importantly, mir-19b existed in all of 3’UTR of *PTENps* and *PTEN* of NMR (Table 2). In addition, the NMR had eight copies of *PTENps* shared three different kinds of MicroRNAs, and four copies of *PTENps* shared two sorts of MicroRNAs. MicroRNA binding sites identified in this study are illustrated in Fig 2.

**Table 2:** The shared specific miRNAs of *PTENs* and *PTENps* 3'UTR.

Name of PTEN	Name of Pesudogene	PTEN and PTENp 3’UTR shared miRNAs
Human PTEN	Human PTENp1	miR-20a,miR-21,miR-214,miR-19b,miR-26a
Chimpanzee PTEN	Chimpanzee PTENp1	miR-20a,miR-21,miR-214,miR-19b,miR-26a
Pygmy chimpanzee PTEN	Pygmy chimpanzee PTENp1	miR-20a,miR-21,miR-214,miR-19b,miR-26a
Gorilla PTEN	Gorilla PTENp1	miR-19b,miR-26a
Baboon PTEN	Baboon PTENp1	miR-20a,miR-21,miR-214,miR-19b,miR-26a
Rhesus macaque PTNE	Rhesus macaque PTENp1	miR-20a,miR-21,miR-214,miR-19b,miR-26a
Crab eating macaque PTEN	Crab eating macaque PTENp1	miR-20a,miR-21,miR-214,miR-19b,miR-26a
Gibbon PTEN	Gibbon PTENp1	miR-20a,miR-21,miR-214,miR-19b,miR-26a
Blind mole rat PTEN	Blind mole rat PTENp1	no shared miRNA
Naked mole rat PTEN	Naked mole rat PTENp1	miR-20a,miR-19b,miR-26a
	Naked mole rat PTENp2	miR-20a,miR-19b,miR-26a
	Naked mole rat PTENp3	miR-20a,miR-19b,miR-26a
	Naked mole rat PTENp4	miR-20a,miR-19b
	Naked mole rat PTENp5	miR-20a,miR-19b,miR-26a
	Naked mole rat PTENp6	miR-20a,miR-19b,miR-26a
	Naked mole rat PTENp7	miR-20a,miR-19b,miR-26a
	Naked mole rat PTENp8	miR-20a,miR-19b,miR-26a
	Naked mole rat PTENp9	miR-20a,miR-19b,miR-26a
	Naked mole rat PTENp10	miR-20a,miR-19b
	Naked mole rat PTENp11	miR-19b
	Naked mole rat PTENp13	miR-20a,miR-19b
	Naked mole rat PTENp14	miR-19b
	Naked mole rat PTENp17	miR-20a,miR-19b
Long tailed chinchilla PTEN	Long tailed chinchilla PTENp1	miR-20a,miR-21,miR-214,miR-19b,miR-26a
Pig PTEN	Pig PTENp1	miR-20a,miR-19b
	Pig PTENp2	miR-20a,miR-19b
	Pig PTENp3	miR-20a,miR-19b,miR-26a
Tasmanian devil PTEN	Tasmanian devil PTENp1	miR-20a,miR-21,miR-19b,miR-26a
Nine banded armadillo PTEN	Nine banded armadillo PTENp1	miR-20a,miR-21,miR-214,miR-19b,miR-26a

## Discussion

In this study, we found that *PTENps* not only existed in the human, but also appeared in some species of primates, rodents, even-toed ungulates, carnivores, cingulata and dasyuromorphia, suggesting that *PTENps* emerged before the divergences of these mammalian orders. However, the majority of other mammals (48 out of 65 mammals) lacked the *PTENp*, which may be due to either the loss of *PTENps* during evolution, or the pseudogenziation of *PTEN* never took place in these species. Since no clade of PTENp genes was shared by all mammalian orders, providing no evidence supporting the origination of *PTENp* before divergence of mammals, it is not clear whether *PTENps* were lost in these 48 species. According to the sequences alignment, we observed that some *PTENps* are completely duplicated from their parental gene and some are partially duplicated, such as the NMR *PTENp*10, 11, 13 and 14. In addition, we found some *PTENps* showed species-specific evolutionary pattern, such as the Tasmanian devil *PTENp*1, nine banded armadillo *PTENp*1 and gibbon *PTENp*1.These results suggest that the mammalian *PTENps* were originated by multiple gene duplications, and experienced the so called ‘birth and death’ evolution[20].

Interestingly, 17 copies of *PTENps* were identified in NMR, which had a high resistance to tumours[21]. Our further MicroRNA binding site prediction results showed that some MicroRNAs can bind with both *PTEN*s and *PTENps* in the NMR and other mammals (Table 2).And most excitingly, we found conserved binding sites for the mir-19b, mir-20a and mir-26a in the 3’UTR of most of the NMR *PTENps* and the *PTEN*. Thus, it is possible that the *PTENps* act as the ceRNAs to regulate the *PTEN* expression in the NMR as well as in the human. A recent study by Abegglen et al. proposed that multiple copies of *TP53* genes in the elephant may help this large animal in resisting cancer[22]. Similarly, the multiple copies of *PTENps* in the NMR may also contribute to its unusual resistance to cancer. But further studies on the expression of *PTENps* and the interaction with miRNAs are needed to support this hypothesis.

However, the BMR, which showed a striking resistance to cancer as well as the NMR[23], is different from the NMR in terms of the copy numbers of *PTENps* and the MicroRNA binding sites. First, we only found one *PTENp* in the BMR compared to17 copies of it in the NMR. Second, unlike in the NMR, no common MicroRNA binding sites were predicted in the 3’UTR of *PTENp*1 and its *PTEN* in BMR. This may indicate that the anticancer mechanism in the BMR is different from that of the NMR. However, the high cut-off value we set may lead to no shared MicroRNA binding sites was found in this study, and it was also possible that the gene-pseudogene crosstalk was mediated by different MicroRNAs in the two species. In addition, some other factors also showed differences between these two species. For example, Fang et al determined that BMR have evolved a cancer-resistance mechanism depending on heightened immunoinflammatory response via gene amplification within the interferon-β1 pathway[23]. But in another study, Tian et al suggested that NMRs had evolved a higher concentration of high-molecular-mass hyaluronan (HA) that restricted cell division when cells gathered closely resulting in cancer resistance[21]. Hence, it is possible that the multiple *PTENps* in NMR function as ceRNAs to regulate its cognate gene by competing for common MicroRNAs, may play an important role in anticancer. Keep in mind, this mechanism may not fit for BMR.

## Conclusions

In conclusion, our findings established that the *PTENps* in mammals originated by multiple gene duplications and experienced the ‘birth and death’ evolution pattern. Some *PTENps* have existed for a long time whereas others have appeared recently. *PTENps* may function as ceRNAs to regulate their *PTEN*s in mammals. Interestingly, the multiples of *PTENps* may compete for the common MicroRNA binding sites in the NMR as well as in the human, which may be responsible for the anticancer trait. These results provide a possible explanation for this anticancer model. However further experiments are needed to prove this hypothesis.

## Supporting Information

S1 Fig TheNeighbour Joining tree of 88 *PTEN*s and *PTENps* in mammals. All bootstrap values were showed.
(EPS)

S2 Fig The Maximum Parsimony tree of 88 *PTEN*s and *PTENps* in mammals. All bootstrap values were showed.
(EPS)

S3 Fig The Maximum Likelihood tree of 88 *PTEN*s and *PTENps* in mammals. All bootstrap values were showed.
(EPS)

S4 Fig The Maximum Parsimony tree of 67 *PTEN*s and *PTENps* in mammals. All bootstrap values were showed.
(EPS)

S5 Fig The Maximum Likelihood tree of 67 *PTEN*s and *PTENps* in mammals. All bootstrap values were showed.
(EPS)

S1 Table The detail information about *PTENs* and *PTENps* in mammals. (XLSX)

S2 Table The detail information about *PTENps* in mammals. (XLSX)

S3 Table The name and sequence information of specific miRNAs. (XLSX)

S4 Table The prediction miRNA binding sites of *PTENs* and *PTENps* 3'UTR in mammals by PITA (The cutoff value is ddG<-3.81). (XLSX)

## References

[1] Jacq C, Miller JR, Brownlee GG. A pseudogene structure in 5S DNA of Xenopus laevis. Cell. 1977; 12 (1): 109–120. 561661 10.1016/0092-8674(77)90189-1

[2] Poliseno L. Pseudogenes: Newly Discovered Players in Human Cancer. Science Signaling. 2012; 5(242): re5 10.1126/scisignal.2002858 22990117

[3] Salmena L, Poliseno L, Tay Y, Kats L, Pandolfi PP. ceRNA hypothesis: The Rosetta Stone of a hidden RNA language? Cell. 2011; 146 (3): 353–358. 10.1016/j.cell.2011.07.014 21802130 PMC3235919

[4] Tay Y, Kats L, Salmena L, Weiss D, Tan SM, Ala U, et al Coding-independent regulation of the tumor suppressor PTEN by competing endogenous mRNAs. Cell. 2011; 147 (2): 344–357. 10.1016/j.cell.2011.09.029 22000013 PMC3235920

[5] Poliseno L, Salmena L, Zhang J, Carver B, Haveman WJ, Pandolfi PP. A coding-independent function of gene and pseudogene mRNAs regulates tumour biology. Nature. 2010; 465 (7301): 1033–1038. 10.1038/nature09144 20577206 PMC3206313

[6] Rutnam ZJ, Du WW, Yang W, Yang X, Yang BB. The pseudogene TUSC2P promotes TUSC2 function by binding multiple microRNAs. Nat Commun. 2014; 5. 10.1038/ncomms3914 PMC3896787 24394498

[7] Esposito F, Martino MD, Petti MG, Forzati F, Tornincasa M, Federico A, et al HMGA1 pseudogenes as candidate proto-oncogenic competitive endogenous RNAs. Oncotarget. 2014; 5 (18): 8341–8354. 10.18632/oncotarget.2202 25268743 PMC4226687

[8] Zheng L, Li X, Gu Y, Lv X, Xi T. The 3'UTR of the pseudogene CYP4Z2P promotes tumor angiogenesis in breast cancer by acting as a ceRNA for CYP4Z1. Breast Cancer Research and Treatment. 2015; 150 (1): 105 10.1007/s10549-015-3298-2 25701119

[9] Karreth F, Reschke M, Ruocco A, Ng C, Chapuy B, Leopold V, et al The BRAF Pseudogene Functions as a Competitive Endogenous RNA and Induces Lymphoma In Vivo. Cell. 2015; 161 (2): 319–332. 10.1016/j.cell.2015.02.043 25843629 PMC6922011

[10] Xing M. Genetic alterations in the phosphatidylinositol-3 kinase/Akt pathway in thyroid cancer. Thyroid. 2010; 20 (7): 697–706. 10.1089/thy.2010.1646 20578891 PMC2935335

[11] Johnsson P, Ackley A, Vidarsdottir L, Lui W, Corcoran M, Grander D, et al A pseudogene long-noncoding-RNA network regulates PTEN transcription and translation in human cells. Nature Structural & Molecular Biology. 2013; 20 (4): 440–446. 10.1038/nsmb.2516 PMC3618526 23435381

[12] Hall TA. BioEdit: a user-friendly biological sequence alignment editor and analysis program for Windows 95/98/NT. Nucleic Acids Symposium Series. 1999; 41: 95–98.

[13] Tamura K, Stecher G, Peterson D, Filipski A, Kumar S. MEGA6: Molecular Evolutionary Genetics Analysis version 6.0. Mol Biol Evol. 2013; 30 (12): 2725–2729. 10.1093/molbev/mst197 24132122 PMC3840312

[14] Kimura M. A Simple Method for Estimating Evolutionary Rates of Base Substitutions Through Comparative Studies of Nucleotide Sequences. 1980. 10.1007/BF01731581 7463489

[15] Kertesz M, Iovino N, Unnerstall U, Gaul U, Segal E. The role of site accessibility in microRNA target recognition. Nat Genet. 2007; 39 (10): 1278–1284.Available from: https://genie.weizmann.ac.il/pubs/mir07/mir07_prediction.html. 10.1038/ng2135 17893677

[16] Kozomara A, Griffithsjones S. miRBase: annotating high confidence microRNAs using deep sequencing data. 2014 Database: miRBase [Internet]. Available from: http://www.mirbase.org/. 10.1093/nar/gkt1181 PMC3965103 24275495

[17] Saitou N, Nei M. The neighbor-joining method: a new method for reconstructing phylogenetic trees. Molecular Biology and Evolution. 1987; 4 (4): 406–425. 3447015 10.1093/oxfordjournals.molbev.a040454

[18] Nei M, Kumar S. Molecular evolution and phylogenetics. Oxford; New York: Oxford University Press; 2000.

[19] Jansson MD, Damas ND, Lees M, Jacobsen A, Lund AH. miR-339-5p regulates the p53 tumor-suppressor pathway by targeting MDM2. Oncogene. 2015; 34 (15): 1908–1918. 10.1038/onc.2014.130 24882579

[20] Piontkivska H, Nei M. Birth-and-death evolution in primate MHC class I genes: divergence time estimates. Mol Biol Evol. 2003; 20 (4): 601–609. 10.1093/molbev/msg064 12679545

[21] Tian X, Azpurua J, Hine C, Vaidya A, Myakishev-Rempel M, Ablaeva J, et al High-molecular-mass hyaluronan mediates the cancer resistance of the naked mole rat. Nature. 2013; 499 (7458): 346–349. 10.1038/nature12234 23783513 PMC3720720

[22] Abegglen L, Caulin A, Chan A, Lee K, Robinson R, Campbell M, et al Potential Mechanisms for Cancer Resistance in Elephants and Comparative Cellular Response to DNA Damage in Humans. JAMA. 2015; 314 (17): 1850–1860. 10.1001/jama.2015.13134 26447779 PMC4858328

[23] Fang X, Nevo E, Han L, Levanon EY, Zhao J, Avivi A, et al Genome-wide adaptive complexes to underground stresses in blind mole rats Spalax. Nat Commun. 2014; 5. 10.1038/ncomms4966 24892994

